# Understanding the Detection Mechanisms and Ability of Molecular Hydrogen on Three-Dimensional Bicontinuous Nanoporous Reduced Graphene Oxide

**DOI:** 10.3390/ma13102259

**Published:** 2020-05-14

**Authors:** Yoshikazu Ito, Megumi Kayanuma, Yasuteru Shigeta, Jun-ichi Fujita, Yoichi Tanabe

**Affiliations:** 1 Institute of Applied Physics, Graduate School of Pure and Applied Sciences, University of Tsukuba, 1-1-1 Tennodai, Tsukuba 305-8571, Japan; fujita@bk.tsukuba.ac.jp; 2 Research Center for Computational Design of Advanced Functional Materials, National Institute of Advanced Industrial Science and Technology, 1-1-1 Umezono, Tsukuba, Ibaraki 305-8568, Japan; megumi.kayanuma@gmail.com; 3 Center for Computational Sciences, University of Tsukuba, 1-1-1 Tennodai, Tsukuba 305-8577, Japan; shigeta@ccs.tsukuba.ac.jp; 4 Department of Applied Science, Okayama University of Science, Okayama 700-0005, Japan; tanabe@das.ous.ac.jp

**Keywords:** porous graphene, graphene oxide, hydrogen adsorption

## Abstract

Environmental safety has become increasingly important with respect to hydrogen use in society. Monitoring techniques for explosive gaseous hydrogen are essential to ensure safety in sustainable hydrogen utilization. Here, we reveal molecular hydrogen detection mechanisms with monolithic three-dimensional nanoporous reduced graphene oxide under gaseous hydrogen flow and at room temperature. Nanoporous reduced graphene oxide significantly increased molecular hydrogen physisorption without the need to employ catalytic metals or heating. This can be explained by the significantly increased surface area in comparison to two-dimensional graphene sheets and conventional reduced graphene oxide flakes. Using this large surface area, molecular hydrogen adsorption behaviors were accurately observed. In particular, we found that the electrical resistance firstly decreased and then gradually increased with higher gaseous hydrogen concentrations. The resistance decrease was due to charge transfer from the molecular hydrogen to the reduced graphene oxide at adsorbed molecular hydrogen concentrations lower than 2.8 ppm; conversely, the resistance increase was a result of Coulomb scattering effects at adsorbed molecular hydrogen concentrations exceeding 5.0 ppm, as supported by density functional theory. These findings not only provide the detailed adsorption mechanisms of molecular hydrogen, but also advance the development of catalyst-free non-heated physisorption-type molecular detection devices.

## 1. Introduction

Detection of molecular hydrogen in ambient atmospheres has become increasingly important because renewable energy, especially hydrogen-based energy, is a focus for the development of sustainable societies [1,2]. Users may encounter some risks associated with the initial leakage of molecular hydrogen from equipment, and explosions can occur with the deployment of molecular hydrogen equipment such as fuel cells. Therefore, the identification of an accurate detection mechanism for molecular hydrogen under hydrogen flow conditions is urgent for the further development of hydrogen gas sensors and the environmental safety of societies that utilize hydrogen. 

Conventional molecules are detected by catalytic combustion, resistance change, and thermal conductivity change [3,4,5,6,7]. Detection is generally accompanied by chemical reactions on the detection element. Heating of the elements may be required for molecular detection. However, such systems might lead to undesirable situations such as spark generation, irreversible exothermic reactions, or overheating, which could occur under abnormal operating conditions in uncontrolled devices. Thus, the possible hazards related to hydrogen gas combustion in conventional gas detection devices cannot be completely excluded. Recently, non-heated and non-chemical reaction detection mechanisms have been reported, employing monolayer or multilayer graphene. These graphene sensors successfully detected gaseous polar molecules such as H_2_O, CO, CO_2_, NO, NO_2_, O_2_, SO_2_, and NH_3_ at parts per billion (ppb) and parts per million (ppm) levels [8,9,10,11,12,13]. Moreover, reduced graphene oxide (rGO) flakes demonstrated highly sensitive polar molecule detection without any catalytic metals [14,15,16,17,18,19], because the molecular adsorption sites of hydroxyl (C-OH), epoxide (C-O-C), ketone (C=O), and carboxyl (COOH) groups on rGO are effective physisorption sites. However, the detection mechanism of non-polar molecules such as molecular hydrogen (H_2_) using either graphene or rGO flakes is not precisely discussed in the literature because the weak physisorption strength and insufficient molecular detection area of the graphene surface mask the details of the electrical resistance behaviors. Since catalytic H_2_ detection with Pt [20,21], Pd [22,23,24,25], Au [26], ZnO [27,28], and SnO_2_ [29,30] at high temperatures has been predominantly employed for detecting H_2_, the significant challenge of realizing physisorption of non-polar H_2_ and revealing the detection mechanisms in detail remains in the development of non-catalytic H_2_ detection devices.

Here, we synthesized a single and monolithic nanoporous reduced graphene oxide (np-rGO) sheet. The synthesized sheet had an edge-free bicontinuous and open porous three-dimensional (3D) structure and was treated by a modified Hummer method. The np-rGO sheet maintained a projected surface area 500 times larger than that of a standard two-dimensional (2D) graphene sheet, performing significantly accurate detection via molecular physisorption of H_2_ as compared to pristine nanoporous unoxidized graphene and rGO flakes. Such physisorption detection mechanisms based on a large surface area provide a new class of design direction for non-heated, flow-type, and physisorption-type graphene-based detection systems for non-polar gas molecules without metal catalysts.

## 2. Materials and Methods 

### 2.1. Preparation of Nanoporous Ni Substrates 

Ni_30_Mn_70_ ingots were prepared by melting pure Ni and Mn (purity >99.9 at.%) in an Ar-protected arc melting furnace. After annealing at 900 °C for 24 h for microstructure and composition homogenization, the ingots were cold-rolled into thin sheets (50 μm) at room temperature. Nanoporous Ni was prepared by chemical dealloying in a 1.0 M (NH_4_)_2_SO_4_ aqueous solution at 50 °C overnight. After dealloying, the samples were rinsed thoroughly with distilled water and then rinsed with ethanol before vacuum drying.

### 2.2. Preparation of Nanoporous Graphene

The nanoporous Ni substrates were loaded in a quartz tube (Ø26 × Ø22 × 250 mm), placed in a quartz tube furnace (Ø30 × Ø27 × 1000 mm), and annealed at 800 °C under a mixed gas flow (200 sccm Ar and 100 sccm H_2_) for 3 min [31,32,33,34,35]. After the reduction pre-treatment, a mixed atmosphere of H_2_ (100 sccm), Ar (200 sccm), and benzene (0.5 mbar, 99.8%, anhydrous, Aldrich) was introduced for graphene growth at 800 °C for 2 min. Subsequently, the furnace was opened for the rapid cooling of the inner quartz tube. The nanoporous Ni substrate was etched in a 1.0 M HCl solution and then the remaining nanoporous graphene was transferred into a 2.0 M HCl solution to completely remove residual Ni. The samples were repeatedly washed with water and subsequently kept in water.

### 2.3. Preparation of Nanoporous Graphene Oxide and Nanoporous Reduced Graphene Oxide

The nanoporous graphene dispersed in water was oxidized via a modified Hummer method [36]. A single graphene sheet was added to a KMnO_4_ (0.45 g), concentrated H_2_SO_4_ (3.45 mL), and NaNO_2_ (0.075 g) mixture without stirring. After 10 min, the mixture was diluted in an aqueous H_2_SO_4_ (5%) solution. Then, an aqueous H_2_O_2_ (30%) solution diluted in aqueous H_2_SO_4_ (5%) was slowly added to the mixture. The obtained transparent sheet was then washed several times with a mixture of aqueous H_2_SO_4_ (3%) and H_2_O_2_ (0.5%). The nanoporous graphene oxide (GO) sheet was then repeatedly washed with water and kept in 2-propanol. The samples were dried by a conventional supercritical CO_2_ drying method. The dried GO sample was transferred onto a SiO_2_ substrate and then connected to fine Cu lines (twisted) using silver paste in a standard pseudo four-probe configuration. The reduced graphene oxide (rGO) sample was prepared by annealing the GO sample on the substrate at 150 °C using ribbon heaters under vacuum in a self-made atmosphere-controlled glass cell connected to Swagelok vacuum components.

### 2.4. Supercritical Drying Procedure

To prevent the collapse of the fragile nanoporous structures caused by water capillary forces during drying, the nanoporous graphene and rGO samples immersed in 2-propanol were dried by using supercritical CO_2_ (scCO_2_) and thus 2-propanol was substituted for CO_2_ without the action of capillary forces. To this end, the nanoporous graphene sample was firstly transferred to a vial (volume: 5 mL) filled with 2-propanol (400 μL), and then the bottle was placed in an 80-mL pressure-resistant container (TAIATSU techno Corp). After removing the air inside the container via CO_2_ purging, the pressure of the container was gradually increased to 15 MPa by introducing liquid CO_2_ (5 Mpa, −4 °C, density: 0.964 g mL^−1^) at a flow rate of 20 mL min^−1^ (~19 g min^−1^) using a high-pressure plunger pump (NP-KX-540, NIHON SEIMITSU KAGAKU Co. Ltd.). The scCO_2_ drying process was performed at 70 °C and at a constant CO_2_ flow rate of 5 mL min^−1^ (~4.8 g min^−1^) by forming a homogeneous phase of 2-propanol and scCO_2_, thus minimizing the capillary force. The pressure was maintained at 15 MPa for 5 h during the drying process. After drying the sample completely, the temperature was set at 40 °C and the container was gradually depressurized over 43 h from 15 MPa to atmospheric pressure by gradually pumping CO_2_ from the system. 

## 3. Microstructure Characterization and Property Measurements

### 3.1. Imaging and Spectroscopic Characterization

The microstructures of the nanoporous GO and nanoporous rGO were characterized using a scanning electron microscope (SEM; JEOL JSM-6700). Chemical analyses on the graphene samples were performed via X-ray photoelectron spectroscopy (XPS; AXIS ultra DLD, Shimazu) with an Al *K*a X-ray monochromator. Raman spectra were obtained using a micro-Raman spectrometer (Renishaw InVia Reflex 532) with an incident wavelength of 532.5 nm. The laser power was set at 0.1 mW to avoid possible damage or unexpected reduction by laser irradiation. The graphene samples were placed on a background-free glass slide. The accumulation time of each spectrum was 100 s. 

### 3.2. Transport Property Measurements

The temperature and magnetic field dependences of the electrical resistance of reduced nanoporous graphene oxide were measured using the four-probe method under a magnetic field at 300 K. The reduced nanoporous graphene sheets were placed on a SiO_2_/Si (SiO_2_: 300 nm) substrate. The electrode was fabricated using Ag epoxy (H20E, Epoxy Technology). The electrical resistance measurements were performed using the Physical Properties Measurement System (PPMS; Quantum Design).

### 3.3. Gas Detection Device Fabrication and Measurements

A molecular detection glass cell was fabricated using an atmosphere-controlled separable three-neck glass cell with an O-ring. Swagelok vacuum components were inserted into two rubber stoppers, and the stoppers were installed in two of the necks. The Swagelok vacuum components were connected to mass flow controllers and a rotary pump. Four Cu wires (1.5 mm) and a thermocouple (type K) were inserted in a rubber stopper, and the stopper was installed in the third neck of the cell to communicate the inside and outside of the cell and the device; all connections were completely sealed with Torr Seal^®^ to avoid leakage. The detection element of the nanoporous GO sheet (7.2 mm × 2.5 mm) was attached on a SiO_2_ substrate through fine twisted Cu lines fixed with silver paste. Then, the substrate was transferred to the glass cell and the fine Cu lines were electrically connected to the Cu wires (1.5 mm) inside the cell. The temperature inside the cell was controlled by an external ribbon heater for thermal reduction of nanoporous GO in the cell. The whole system and the connected vacuum components were heated to 150 °C, and the GO (already connected to the Cu wires (1.5 mm) through the fine Cu wires) was thermally reduced to rGO under vacuum (5 Pa). Briefly, the temperature for thermal reduction was fixed at 150 °C and the heating time was set at 4 h for oxygen contents of 20.0 at.%, 6 h for oxygen contents of 18.2 at.%, and 12 h for oxygen contents of 10.8 at.%. After cooling down to the room temperature (23 °C), the thermally reduced GO samples were sequentially measured in the cell by a standard pseudo four-probe method using a semiconductor parameter analyzer (Keysight B1500A). The applied voltage was set at −9 V for the nanoporous rGO samples (with chemical oxidation and thermal reduction treatments) and to −10 mV for the pristine nanoporous graphene (without oxidation and reduction treatments) under a mixture of gaseous argon (Ar: >99.9999 vol.%, H_2_O: <−80 °C, O_2_: <0.1 ppm, CH_4_: <0.1 ppm, CO_2_: <0.1 ppm, CO: <0.1 ppm, N_2_: <0.6 ppm, H_2_: <0.1 ppm). The ultrapure gaseous Ar continuously flowed until the resistance value stabilized to exclude unexpected influences of impurities such as H_2_O, O_2_, CH_4_, CO_2_, CO, and H_2_. Thus, any changes in resistance values under the mixture of H_2_/Ar gas flowing could be attributed to the introduction of ultrapure H_2_ gas. After the resistance was stable, the mixture of gaseous Ar and gaseous hydrogen (H_2_: >99.9999 vol.%, H_2_O: <−80 °C, O_2_: <0.1 ppm, CH_4_: <0.1 ppm, CO_2_: <0.1 ppm, CO: <0.1 ppm) was introduced in the cell. The total flow rate of the mixed gas was set at 30 sccm. To obtain the 1 ppm H_2_ gas in the Ar flow case, gaseous hydrogen was mixed with gaseous argon to a H_2_ concentration of 0.0001 vol.%, and then the mixed gas was supplied by the mass flow controllers at a flow rate of 30 sccm (the partial pressure of hydrogen was 0.10 Pa). Note that flow rates exceeding 30 sccm deformed the atomically thin porous structures, and the resistance values became unstable. Before all measurements, the whole system was vacuumed for 12 h at room temperature to avoid further thermal reduction of the rGO. 

## 4. Density Functional Theory Calculations 

The geometries of the surface models (C_54_H_18_, C_54_H_18_-2OH, C_54_H_18_-2O, C_53_H_18_-COOH, and C_53_H_18_-2O) were optimized using the density functional theory (DFT) method with the B3LYP functional [37,38] and considering the D3 version on Grimme’s dispersion correction [39] with 6-311G(d,p) basis sets [40]. For the graphene oxide models, the coordinates of the terminal H atoms were fixed to those of the pristine graphene to model a large surface that would prevent distortion. Several structures from the graphene oxide models were compared and the most stable alternative was chosen for the calculation of H_2_ adsorption. All calculations were performed using Gaussian 16 quantum chemistry software [41].

## 5. Results

### 5.1. Material Characterization

**Figure 1:**
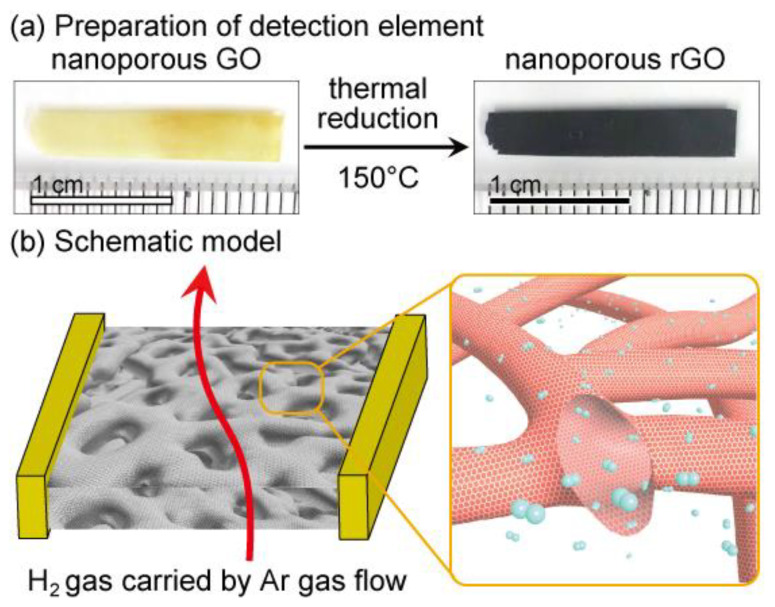
Nanoporous reduced graphene oxide. (**a**) Optical images of nanoporous graphene oxide (GO) and nanoporous reduced GO (rGO), and (**b**) a schematic illustration of the detection element of nanoporous rGO with details. The inset shows the tubular graphene ligaments.

**Figure 2:**
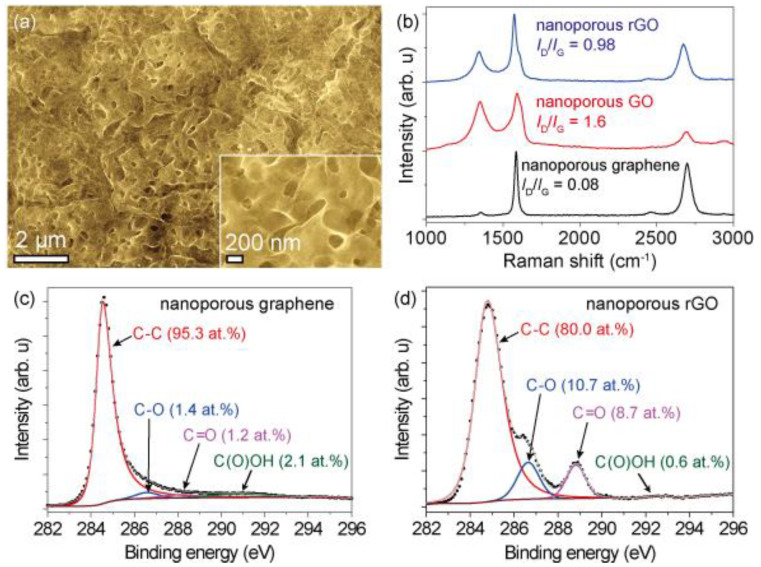
Characterizations of nanoporous rGO. (**a**) SEM images of nanoporous rGO with an oxygen content of 20.0 at.%. (**b**) Raman spectra of pristine nanoporous graphene, nanoporous GO, and nanoporous rGO. X-ray photoelectron spectra of C 1s for (**c**) pristine nanoporous graphene and (**d**) nanoporous rGO with an oxygen content of 20.0 at.%.

The transparent 3D np-GO (Figure 1a) was dried by a conventional supercritical CO_2_ fluid method, attached to a SiO_2_ substrate with the Cu wires (Figure 1b), and transferred to the atmosphere-controlled glass cell to reduce the np-GO sample at 150 °C for 4 h under vacuum. The dried np-rGO maintained the original morphology of the pristine 3D nanoporous graphene without any obvious flakes or holes after the oxidation and reduction processes (Figure 2a and Figure S1). The specific surface area and average pore size were ~1000 m^2^ g^−1^ and ~200 nm, respectively, and the total projected surface area exceeded 500 times that of the 2D graphene sheet [32,36,42]. Moreover, it was determined by high-resolution transmission electron microscopy that amorphous-like graphene lattices on np-rGO were partially crystallized after thermal reduction, and electron energy-loss spectroscopy (EELS) spectra also demonstrated regeneration of *sp*^2^ bonds by post reduction [36]. EELS mappings confirmed that the oxidation species were almost homogeneously distributed on the nanoporous structures [36].

Raman spectra were analyzed to estimate the quality of the graphene samples and their respective oxidation levels (Figure 2b and Figure S2 and Table S1). The pristine nanoporous graphene showed an intensity ratio of the 2D and G bands (*I*_2D_/*I*_G_) of 2.5 and of the D and G bands (*I*_D_/*I*_G_) of 0.08, corresponding to high-quality 1–2 layer graphene without detectable amorphous carbon features [43,44]. After the oxidation process, the np-GO showed a large defect density (*I*_D_/*I*_G_ of 1.6 and *I*_2D_/*I*_G_ of 0.91). The np-rGO partially restored graphene features (*I*_D_/*I*_G_ of 0.98 and an *I*_2D_/*I*_G_ of 1.7). The full width at half maximum (FWHM) values of the D, G, and 2D bands of the np-rGO were significantly increased in comparison to the pristine nanoporous graphene. Chemical binding was further investigated by XPS. The C1s X-ray photoelectron spectrum (Figure 2c,d, Figures S3 and S4) demonstrated an oxygen content of 4.7 at.% for pristine nanoporous graphene, 32.8 at.% for np-GO, and 20.0 at.% for np-rGO. Oxidized carbon species such as 10.7 at.% C-O and/or C-O-C (286.6 eV), 8.7 at.% C=O (288.8 eV), and 0.6 at.% C(O)OH (290 eV) were observed on the np-rGO, which was consistent with reported observations [36,45,46,47]. The FWHM of graphitic carbon at 284.5 eV was broadened from 0.71 eV of pristine nanoporous graphene to 2.0 eV of np-GO after oxidation and narrowed to 1.3 eV of np-rGO after reduction; this was associated with the mixed states of orbitals *sp*^2^ and *sp*^3^ [46,47]. Therefore, the np-rGO presented a significant number of defect structures containing C-O, C-O-C, and C=O with a small number of C(O)OH. Moreover, it was confirmed that the X-ray photoelectron spectra of Ni 2p and Mn 2p were not detected in any of the samples (Figures S3 and S4), which was consistent with the absence of Ni and Mn compounds in the X-ray diffraction (XRD) spectrum (Figure S5). Other np-rGO samples with different oxygen contents were similarly characterized (Figures S2–S4). Furthermore, the inter-defect distance on the graphene lattice estimated by Raman spectra and the distance between functional groups estimated by XPS were compared. The average inter-defect distance was estimated by the *I*_D_/*I*_G_ from the Tuinstra–Koenig relation: *I*_D_/*I*_G_ = *C*(λ)/*L*_D_, where *C*(λ) is a proportionality constant at the excitation laser wavelength λ and *L*_D_ is the average distance between the defects [48,49,50]. Under the assumption that this relation could be applied to the rGO system, the *L*_D_ value of np-rGO with an oxygen content of 20 at.% (*I*_D_/*I*_G_ = 0.98) was approximately 8–10 nm, which was almost of the same order as the distance between the oxidized functional groups estimated by the atomic concentrations.

The transport properties of nanoporous graphene and np-rGO were measured at 300 K by a standard four-probe method. The typical electrical conductances and electron mobilities of the nanoporous graphene samples were 500–1000 S cm^−1^ and 5000–8000 cm^2^ V^−1^ s^−1^, respectively [35,51]. The typical electrical conductances and electron mobilities of the np-rGO samples with degrees of oxidation of 19.6–23.3 at.% were 0.0025–0.043 S cm^−1^ and 0.31–1.0 cm^2^ V^−1^ s^−1^, respectively [36]. Note that the electron mobility of the np-rGO samples was ca. 2 orders of magnitude higher than that of conventional rGO films formed by small flakes under the same thermal reduction conditions (0.0015–0.06 cm^2^ V^−1^ s^−1^) [52]. Therefore, the electrons on np-rGO were moved smoothly owing to the unique bicontinuous structures.

### 5.2. Hydrogen Adsorption 

**Figure 3:**
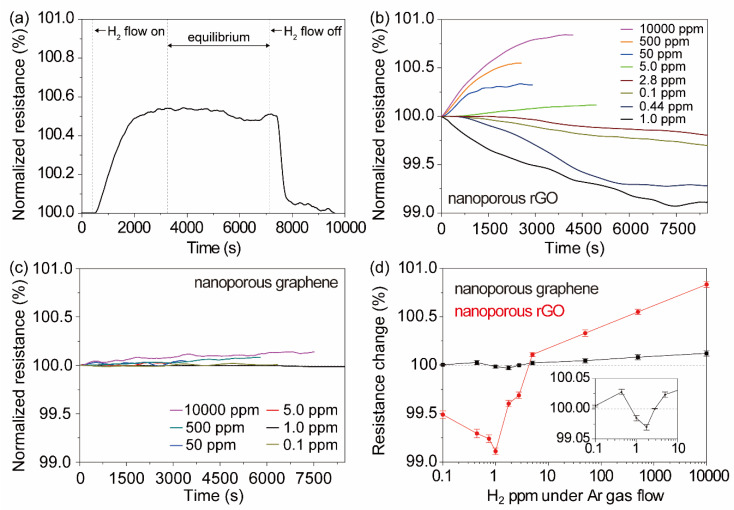
Hydrogen detection ability of nanoporous rGO under H_2_/Ar gas flow at 1 atmosphere. (**a**) Reversible feature of nanoporous rGO. Time dependence of resistance change (%) on (**b**) nanoporous rGO and (**c**) pristine nanoporous graphene. (**d**) H_2_ volume concentration (ppm) dependence of resistance change (%) of nanoporous rGO with an oxygen content of 20.0 at.% and pristine nanoporous graphene. Error bars were estimated by fluctuations during measurements.

To evaluate the molecular physisorption ability of H_2_, we measured the time dependence of resistance changes under the H_2_/Ar mixed gas in the atmosphere-controlled glass cell. Firstly, we confirmed the reversibility of molecular physisorption of H_2_ under Ar and H_2_/Ar flow conditions (500 ppm H_2_, Figure 3a) on the np-rGO with an oxygen content of 20.0 at.%. In the first step, pure Ar gas flowed until the resistance value became constant, and then H_2_/Ar gas flowed; time was necessary to replace the pure Ar gas in the cell with H_2_/Ar gas. The resistance values gradually increased as the H_2_ reached the surface of the np-rGO. The resistance values kept substantially constant at the equilibrium states of adsorption and desorption of H_2_ under the H_2_/Ar gas flow. The resistance values gradually decreased to the initial resistance value after stopping the H_2_ gas supply (i.e., 30 sccm pure Ar gas flow only). The response was relatively slower than that of conventional catalytic metal-based gas detection systems, probably because the replacement of Ar by H_2_ requires time owing to the low flow rate and geometrically requires time to reach the surface due to the complicated porous structure [32] in comparison to the flat structures [20,21,22,23,24,25,26,27,28,29,30].

The change in resistance at H_2_ gas volume concentrations from 0.1 ppm to 10,000 ppm under H_2_/Ar gas flow (30 sccm in total) was investigated for the np-rGO with an oxygen content of 20.0 at.% (Figure 3b and Figure S6) and nanoporous graphene (Figure 3c). We found that the time-dependent resistance values at −9.0 V of the np-rGO (initial resistance: 742 MΩ) increased above 5 ppm H_2_ under H_2_/Ar gas flow, whereas they decreased below 2.8 ppm H_2_. Each change of resistance value was normalized to the equilibrium state before H_2_ adsorption (only Ar flow state) and plotted for the various H_2_ ppm values (Figure 3d). The normalized changes (%) were proportional to logarithmic H_2_ ppm values at above 5 ppm H_2_ under H_2_/Ar gas flow. The nanoporous graphene (initial resistance: 33.8 Ω) showed a similar behavior but did not show large changes in resistance values for 0.1–10,000 ppm H_2_ under the same conditions. Thus, the large changes in np-rGO were attributed to the oxidized functional groups on the graphene. Moreover, both samples exhibited a local minimum at approximately 1 ppm H_2_ under H_2_/Ar gas flow. Note that these increases and decreases have not been reported for graphene and rGO detection systems [8,14,15,16,17,18,19]. Furthermore, the oxidization level dependence of the resistance changes was similarly investigated (Figure S7). The np-rGO samples with oxygen contents of 10.8 at.% (initial resistance: 11.1 kΩ) and 18.2 at.% (initial resistance: 1.26 MΩ) showed similar behaviors, but smaller resistance changes at typical H_2_ ppm values under H_2_/Ar gas flow in comparison to the np-rGO sample with an oxygen content of 20.0 at.%. 

### 5.3. DFT Calculations

**Figure 4:**
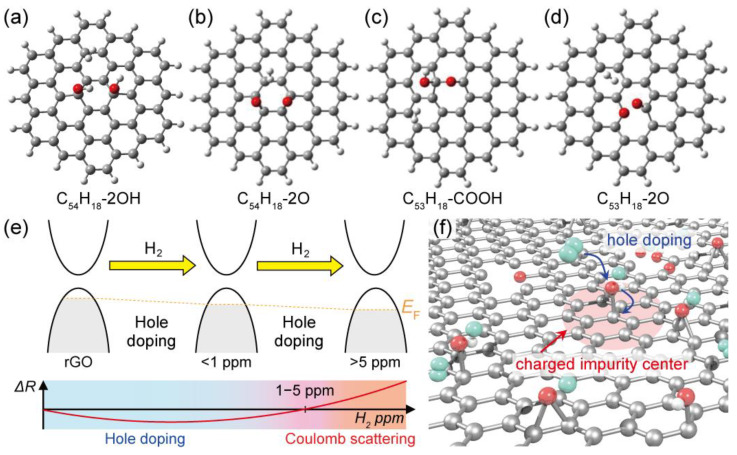
Density functional theory (DFT)-calculated adsorption of molecular hydrogen on graphene oxide and mechanism of resistance change. Graphene oxide with (**a**) two hydroxyl groups, (**b**) two epoxide groups, (**c**) a carboxyl group, and (**d**) ether and ketone groups after adsorption of molecular hydrogen. (**e**) Charge transfer effect and electronic density of states on the nanoporous rGO before and after H_2_ adsorption with expected resistance changes. (**f**) Coulomb scattering effect on nanoporous rGO after adsorption. The oxygen-containing functional groups act as charged impurity centers enhanced by hole doping from the adsorbed H_2_.

To elucidate the resistance behaviors, we performed DFT calculations for molecular physisorption of H_2_ on the surface of graphene and graphene oxide (Figure 4a–d and Figure S8 and Table 1). We employed a nanographene cluster model with C_54_H_18_ and partly oxidized the nanographene to introduce oxygen-containing functional groups such as hydroxyl, epoxide, ether, ketone, and carboxyl groups for the adsorption of a single H_2_ molecule (Figure S9). The adsorption energy of H_2_ was calculated in these models and was −3.14 kcal/mol for C_54_H_18_ with two hydroxyl groups, −2.73 kcal/mol for C_54_H_18_ with two epoxide groups, −2.72 kcal/mol for C_54_H_18_ with a carboxyl group, and −2.88 kcal/mol for C_53_H_18_ with an ether and a ketone group; these values were lower than that for C_54_H_18_ without any functional groups (i.e., −1.58 kcal/mol), and there were no significant differences in the functional groups. In the models, it was found that two kinds of charge transfer phenomena occurred: (1) from oxygen-containing functional groups to graphene oxide (i.e., hole doping initially occurred from oxygen-containing functional groups to graphene) and (2) from a single H_2_ molecule to graphene oxide with +0.020 e (i.e., additional hole doping occurred from H_2_ to graphene) (Figure 4 and Figure S10 and Table 1). This suggests that the np-rGO before adsorption was electron-deficient because of the oxidized functional groups and became further electron-deficient after H_2_ adsorption. Moreover, the energy levels of the highest occupied molecular orbital (HOMO) and lowest unoccupied molecular orbital (LUMO) decreased after the adsorption (Table 1). In particular, the energy level of the LUMO of the C_54_H_18_ with two hydroxyl groups showed the highest reduction, in which the adsorption of H_2_ modified the electronic state of the rGO, benefitting the sequential adsorption of additional H_2_. Notably, we found a sequential adsorption of three additional H_2_ molecules by the C_54_H_18_ model with two hydroxyl groups (−10.90 kcal/mol in total) and a charge transfer (+0.074 e by four H_2_ molecules) from the additional H_2_ molecule on graphene oxide further occurred (Figure S11 and Table S2).

## 6. Discussion

The concentrations of adsorbed H_2_ lower than 1 ppm showed negative resistance changes, while those higher than 5 ppm showed positive resistance changes. The high resistance changes are attributed to the high oxidation level of the samples (Figure S7) and lower than 1 ppm or higher than 500 ppm H_2_ concentrations (Figure 3 and Figure S7). This suggests that low amounts of adsorbed H_2_ should promote the charge transfer effect (Table 1), while high amounts of adsorbed H_2_ should present a larger influence on resistance change and promote an additional charge transfer effect on the oxidized functional groups (Table S2). Combining the experimental results and DFT calculations, two mechanisms of resistance change could arise for the threshold amount of 1–5 ppm H_2_ under H_2_/Ar gas flow (30 sccm in total). Below 1 ppm H_2_, contribution of Coulomb scattering by the adsorbed H_2_ was not dominant/apparent and the charge transfer from H_2_ to np-rGO was a major effect. In this case, further hole doping from the adsorbed H_2_ of the p-type np-rGO reduced the resistance (Figure 4e). Conversely, above 5 ppm H_2_, Coulomb scattering was the dominant effect, and charge transfer from H_2_ to np-rGO was a minor effect. The resistance was simply enhanced by the increase of charged impurity centers (Figure 4e,f), in accordance with a previous report [9]. Notably, even for 5 ppm H_2_, it was observed that the resistance firstly decreased from 0 s to 300 s (charge transfer domination) and gradually increased after 300 s (Coulomb scattering domination) with an increase in hydrogen concentration (Figure S12). Thus, in the range from 1 to 5 ppm H_2_, the Coulomb scattering and charge transfer effects may be comparable, giving rise to a local minimum. Moreover, the DFT calculations suggest that the hydroxyl groups play an important role in the sequential adsorption of a large amount of H_2_. The np-rGO with an oxygen content of 20.0 at.% (hydroxyl/epoxide group: 10.7 at.%) showed the largest change of resistance values at higher H_2_ ppm values in comparison to the nanoporous graphene and np-rGO with oxygen contents of 10.8 at.% (hydroxyl/epoxide group: 2.5 at.%) and 18.2 at.% (hydroxyl/epoxide group: 6.3 at.%). Furthermore, open porous structures with large total surface areas whose surface was modified by the oxygen-containing functional groups significantly promoted mass transfer and increased the sensitivity of molecular physisorption of non-polar H_2_. The excellent transport properties resulting from such monolithic characteristics supported accurate detection of resistance changes.

## 7. Conclusions

We developed a novel 3D nanoporous rGO for molecular hydrogen detection at room temperature under gas flow conditions. The detection mechanisms of molecular hydrogen were revealed by using 3D nanoporous rGO specimens featuring a large surface area, a single bicontinuous monolithic sheet morphology, and unique transport properties (non-electron hopping conduction), and were found to be based on the charge transfer effect and Coulomb scattering effect. Moreover, DFT calculations revealed that hydroxyl, epoxide, ether, ketone, and carboxyl groups are preferable molecular physisorption sites, and that hydroxyl groups benefit from the sequential adsorption of molecular hydrogen. Nanoporous rGO with tunable oxygen-containing functional groups could provide a general platform for a room-temperature physisorption-type gas sensor and provide a new design direction for nanoporous material-based gas sensors for combustible gases.

## Figures and Tables

**Table 1:** Summary of the highest occupied molecular orbital (HOMO) energy level, the lowest unoccupied molecular orbital (LUMO) energy level, the shift of HOMO (Δ*E*_HOMO_) and LUMO (Δ*E*_LUMO_) energy level and cluster total energy before and after adsorption of molecular hydrogen. Charge transfer from H_2_ to GO indicates the sum of the Mulliken charge of the GO model.

	Before Absorption of H_2_		After Absorption of H_2_			
	HOMO (eV)	LUMO (eV)	Δ*E*_HOMO_ (eV)	Δ*E*_LUMO_ (eV)	Δ*E*_abs_ (kcal/mol)	Charge Transfer from H_2_ to GO
C_54_H_18_	−0.19069	−0.08710	−0.0002	−0.0001	−1.58	−0.007 e
C_54_H_18_-2OH	−0.19323	−0.09767	−0.0004	−0.0007	−3.14	+0.023 e
C_54_H_18_-2O	−0.19819	−0.09185	−0.0004	−0.0005	−2.73	+0.020 e
C_53_H_18_-COOH	−0.17395	−0.12959	−0.0003	−0.0005	−2.72	+0.018 e
C_53_H_18_-2O	−0.18408	−0.09939	−0.0003	−0.0007	−2.88	+0.019 e

## Supplementary Materials

The following are available online at https://www.mdpi.com/1996-1944/13/10/2259/s1, Figure S1–S11, Table S2.
