# Supplementary material for: Understanding the Detection Mechanisms and Ability of Molecular Hydrogen on Three-Dimensional Bicontinuous Nanoporous Reduced Graphene Oxide
Source: Materials (Basel). 2020 May 14;13(10):2259. doi: 10.3390/ma13102259 (PMC7288210; doi:10.3390/ma13102259)
Supplement: Supplementary file 1 [file materials-13-02259-s001.pdf]

materials

Supporting Information

Understanding the Detection Mechanisms and Ability of Molecular Hydrogen on Three-Dimensional Bicontinuous Nanoporous Reduced Graphene Oxide

Yoshikazu Ito[1,*], Megumi Kayanuma[2,3], Yasuteru Shigeta[3], Jun-ichi Fujita[1] and Yoichi Tanabe[4]

1 Institute of Applied Physics, Graduate School of Pure and Applied Sciences, University of Tsukuba, 1-1-1 Tennodai, Tsukuba 305-8571, Japan.
2 Research Center for Computational Design of Advanced Functional Materials, National Institute of Advanced Industrial Science and Technology, 1-1-1 Umezono, Tsukuba, Ibaraki 305-8568, Japan.
3 Center for Computational Sciences, University of Tsukuba, 1-1-1 Tennodai, Tsukuba 305-8577, Japan.
4 Department of Applied Science, Okayama University of Science, Okayama, 700-0005, Japan.
* Correspondence: ito.yoshikazu.ga@u.tsukuba.ac.jp

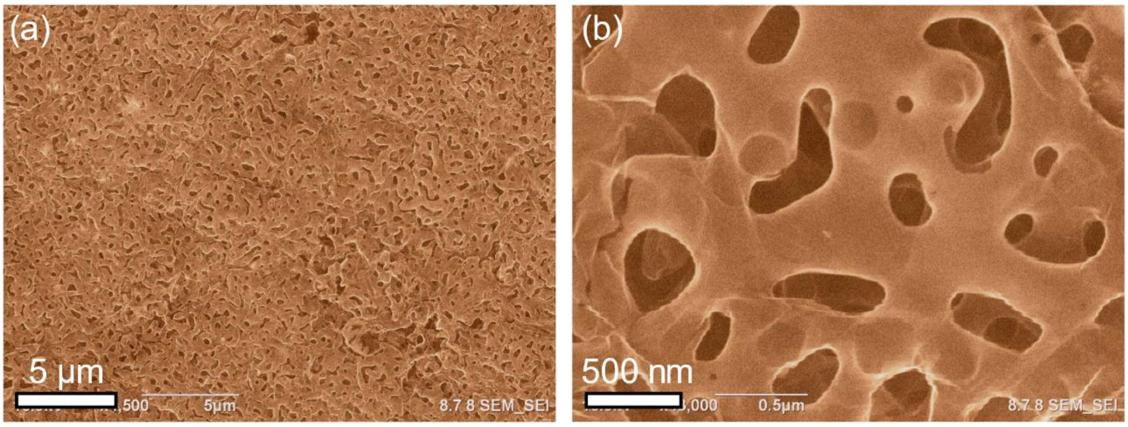

Figure S1. Typical SEM image of high and low magnification of pristine nanoporous graphene[i,ii].

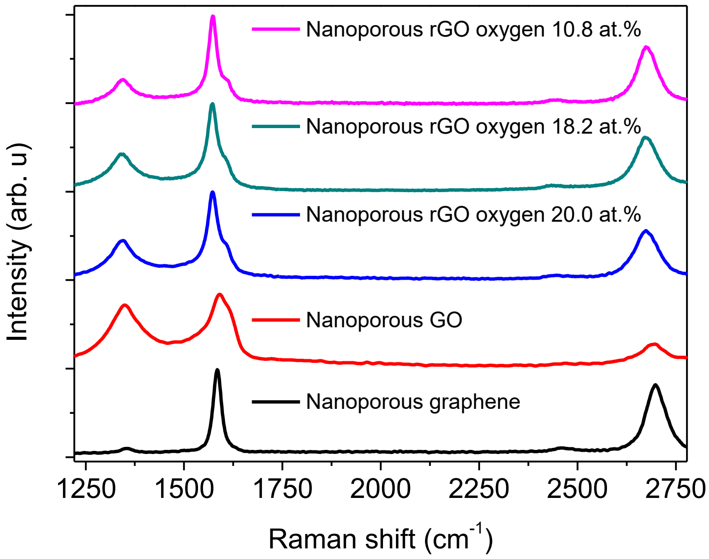

Figure S2. Raman spectra of pristine nanoporous graphene, nanoporous GO, nanoporous rGO with different oxidation contents.

Table S1. Raman band measurements of the nanoporous graphene, nanoporous graphene oxide and nanoporous reduced graphene oxide samples used in the main text. Unit of the spectra and line width is cm^{-1}.

	**D band Line width**	**G band Line width**	**D' band Line width**	**2D band Line width**	I_D/I_G	I_{2D}/I_G
Nanoporous graphene	1353<br>18	1584<br>10	-	2699<br>29	0.084	2.5
Nanoporous GO	1348<br>30	1584<br>18	1615<br>16	2692<br>44	1.6	0.91
Nanoporous rGO oxygen 20.0 at.%	1343<br>26	1571<br>12	1605<br>17	2673<br>35	0.98	1.7
Nanoporous rGO oxygen 18.2 at.%	1342<br>25	1571<br>12	1604<br>17	2674<br>35	0.95	1.8
Nanoporous rGO oxygen 10.8 at.%	1343<br>22	1571<br>12	1605<br>17	2675<br>30	0.51	1.8

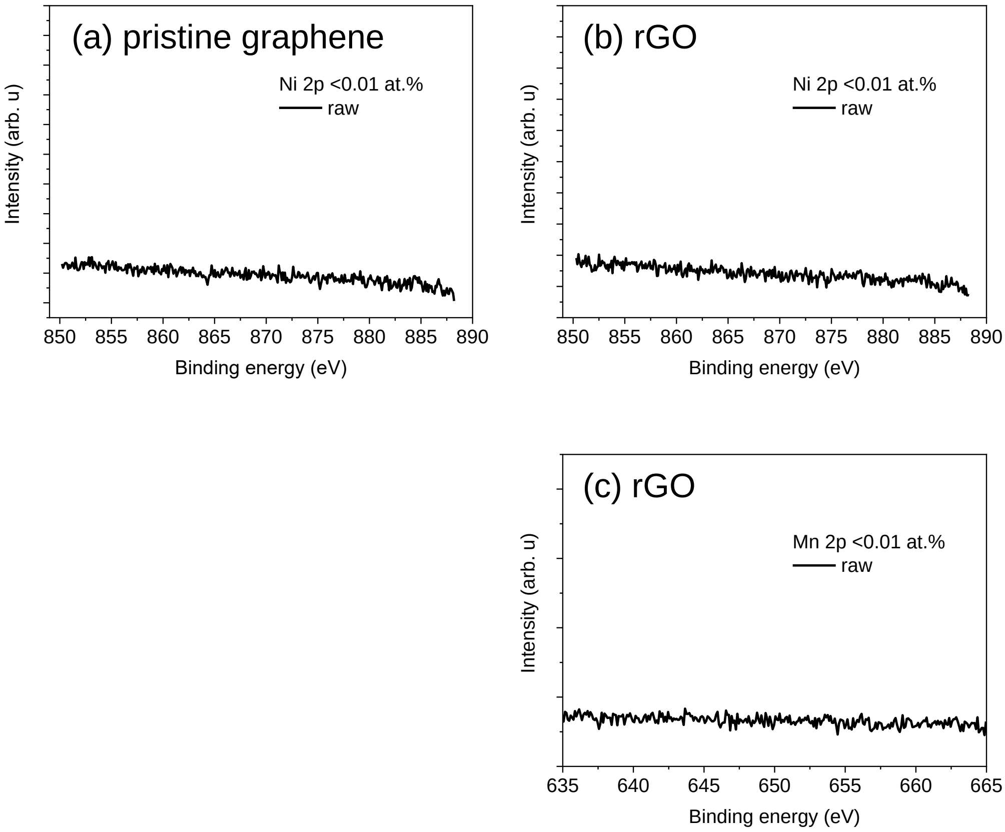

Figure S3. XPS spectra of Ni 2p and Mn 2p in pristine graphene and rGO (oxygen contents of 20 at.%). The residual Ni and Mn atomic concentrations were less than 0.01 at%, respectively.

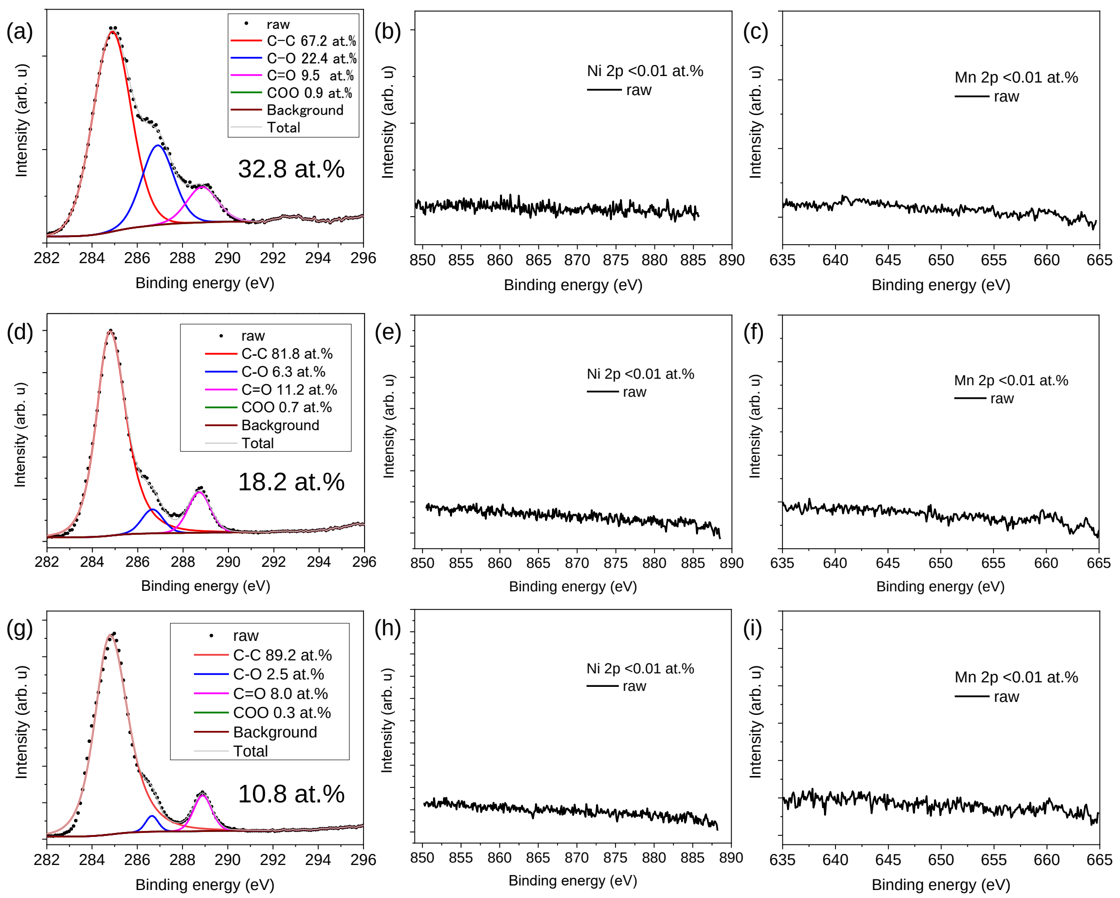

Figure S4. XPS spectra of C 1s, Ni 2p and Mn 2p in (**a-c**) nanoporous GO with oxygen contents of 32.8 at.% and (**d-f**) nanoporous rGO with oxygen contents of 18.2 at.% and (**g-i**) 10.8 at.%. The residual Ni and Mn atomic concentrations were less than 0.01 at%, respectively.

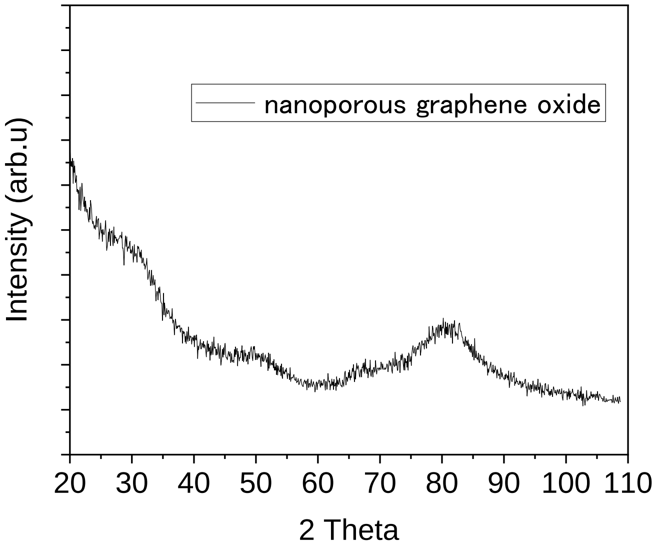

Figure S5. Typical XRD spectra of nanoporous graphene oxide.

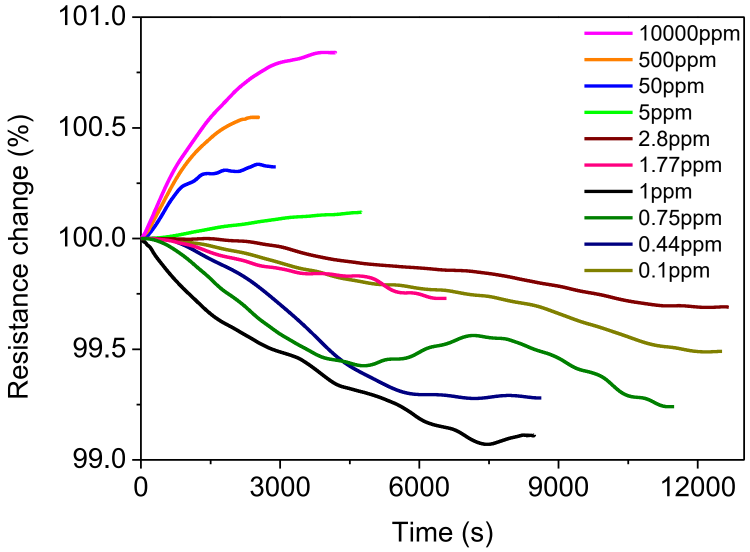

Figure S6. Time dependence of resistance changes with various H_2 volume concentrations under the H_2/Ar gas flow for the nanoporous rGO with oxygen contents of 20.0 at.%.

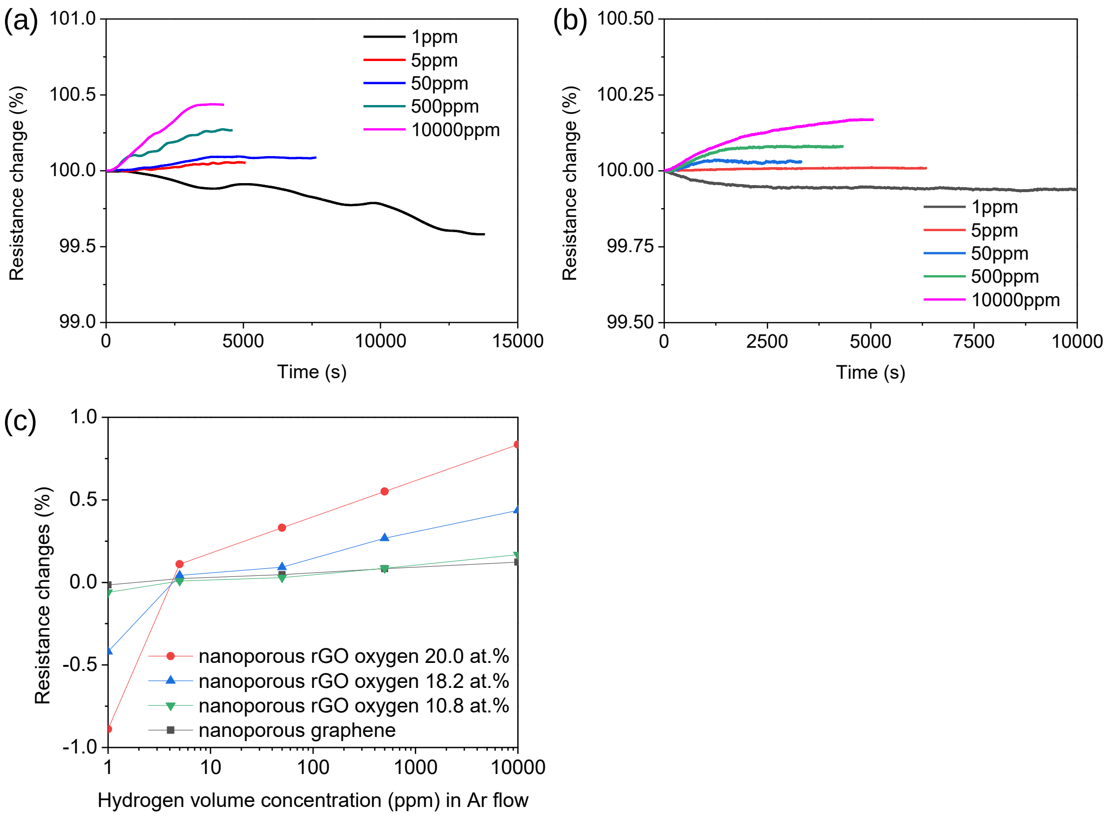

Figure S7. Time dependence of resistance changes with various H_2 volume concentrations under the H_2/Ar gas flow for the nanoporous rGO with oxygen contents of (**a**) 18.2 at.% and (**b**) 10.8 at.% and (**c**) the resistance changes (%).

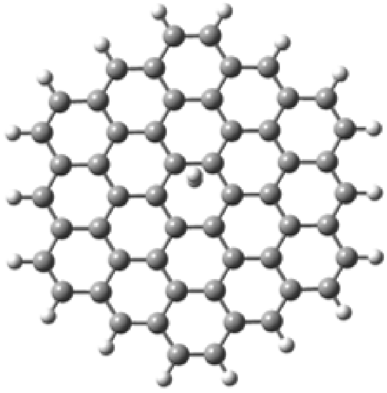

$C_{54}H_{18}$

Figure S8. Graphene model after adsorption of a hydrogen molecule (white ball).

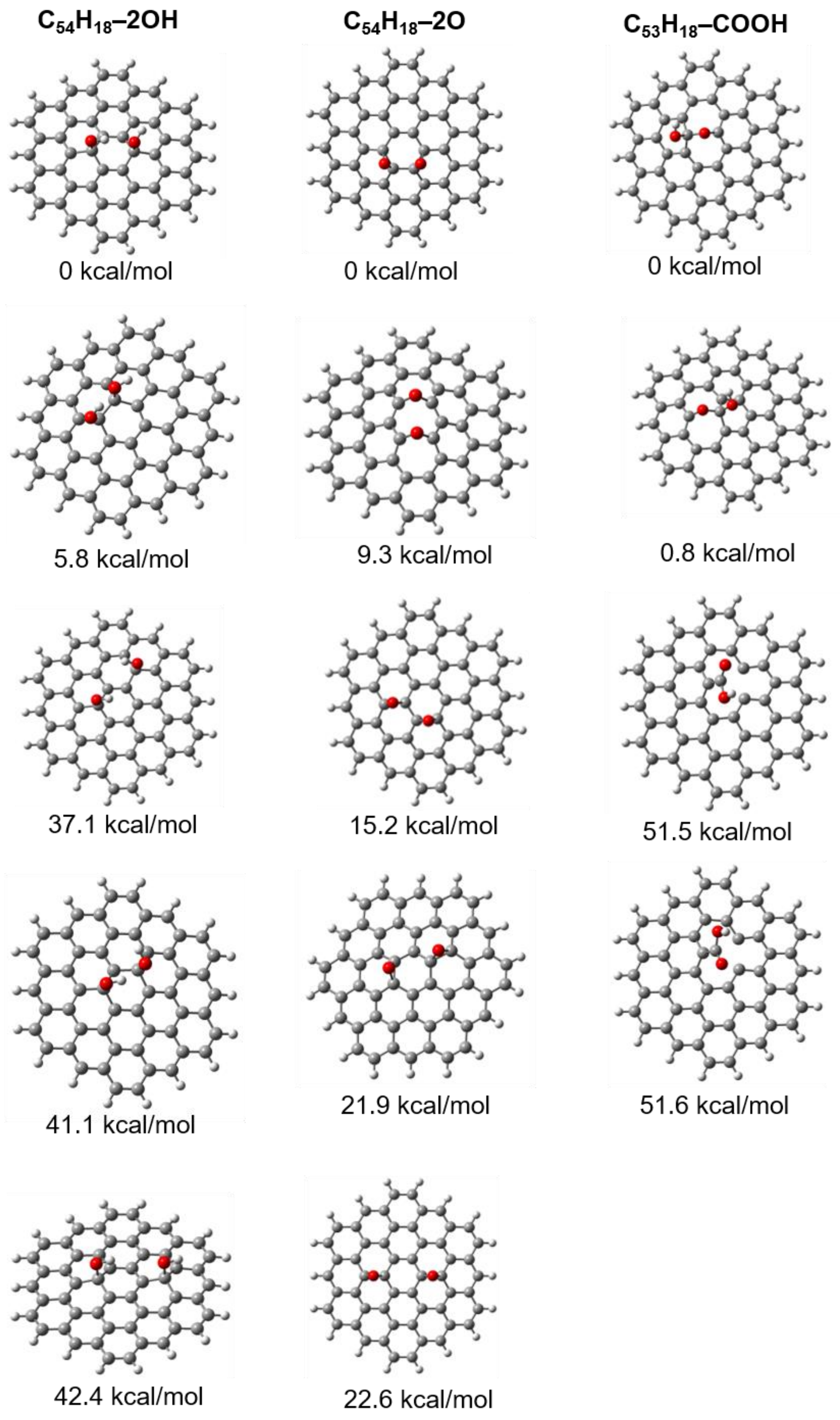

Figure S9. Optimized structures of graphene oxide models with different configuration of functional groups. White and red balls present hydrogen and oxygen atom.

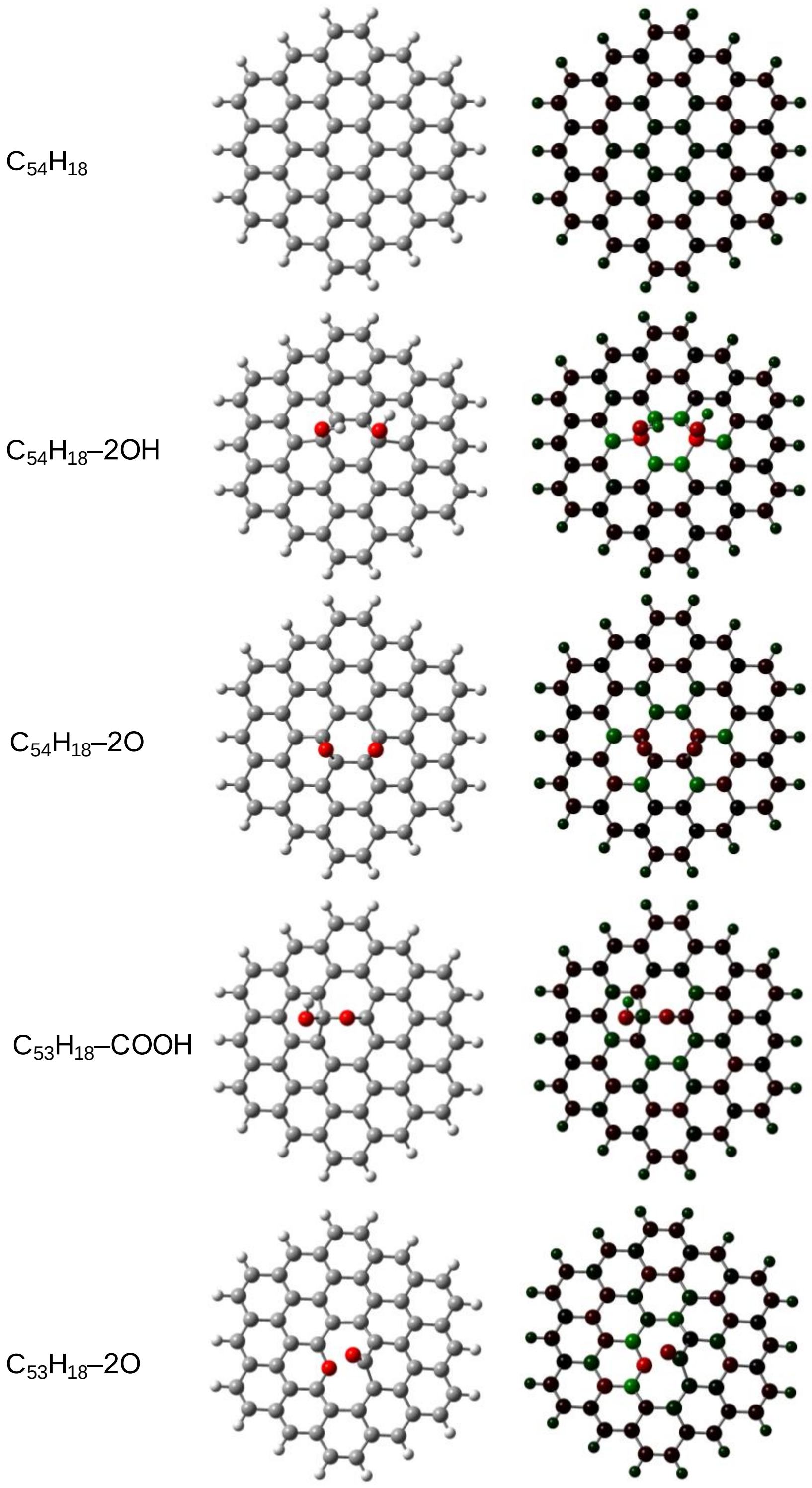

Figure S10. Optimized structure (left, white and red balls present hydrogen and oxygen atom) and Mulliken charge (right) of the model surfaces. Red and green indicate negative and positive charge (color range of −0.7 to 0.7).

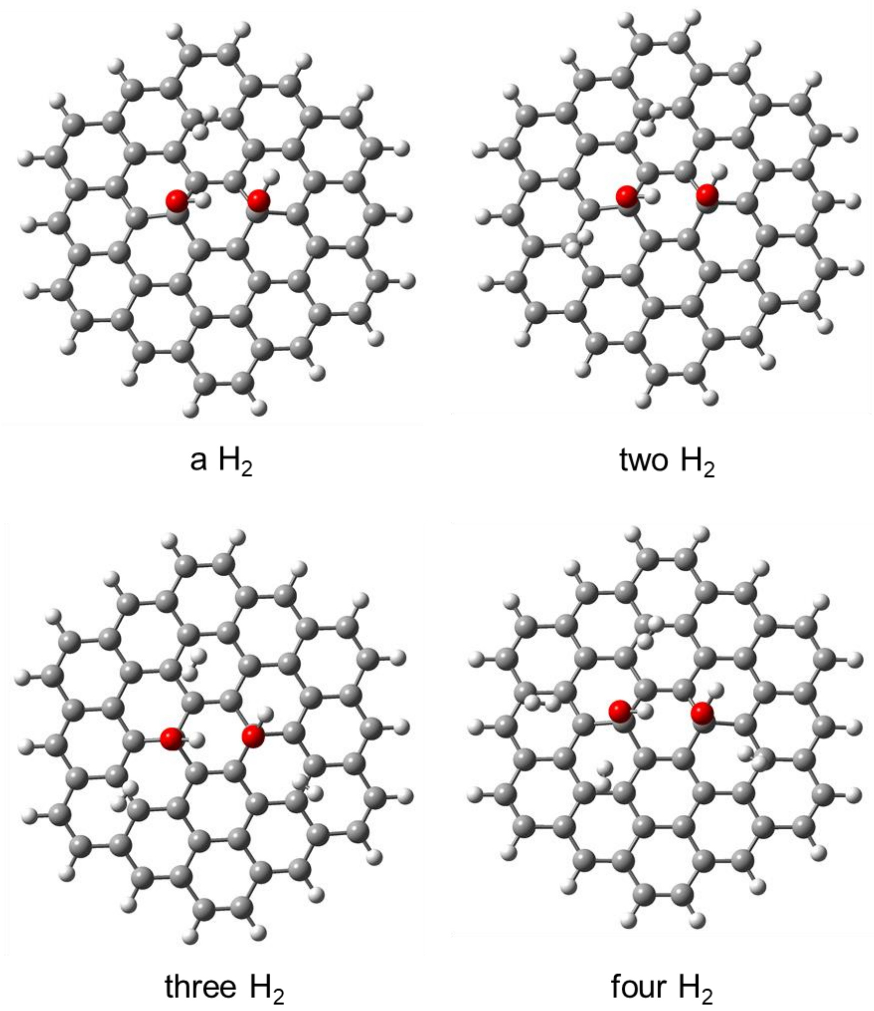

Figure S11. Optimized structure of $C_{54}H_{18}$-2OH after adsorption of H_2. White and red balls present hydrogen and oxygen atom.

Table S2. Summary of the change of HOMO, LUMO, cluster total energy, and charge transfer from each hydrogen molecules to GO after the adsorption of hydrogen molecules.

	ΔE_{HOMO} (eV)	ΔE_{LUMO} (eV)	ΔE_{ads} (kcal/mol)	Charge transfer from H_2 to GO
1 H_2	−0.0004	−0.0007	−3.14	+0.023
2 H_2	−0.0008	−0.0014	−5.81	+0.023, +0.022
3 H_2	−0.0011	−0.0021	−8.43	+0.023, +0.022, +0.023
4 H_2	-0.0013	-0.0025	−10.90	+0.017, +0.019, +0.022, +0.016

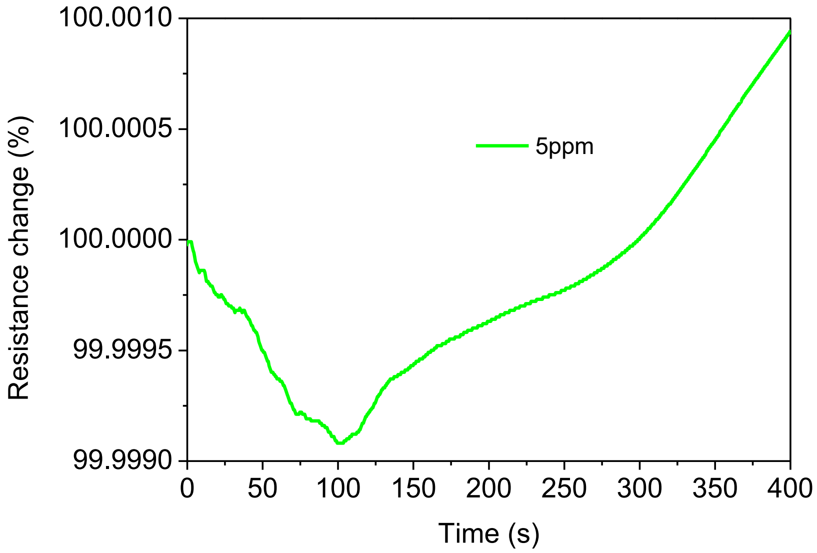

Figure S12. Time dependence of resistance change (%) on the nanoporous rGO (oxygen content of 20 at.%) under 5 ppm H_2. The 0.001 % resistance change is equivalent to 742 kΩ.

References

[i] Y. Ito, Y. Tanabe, H. J. Qiu, K. Sugawara, S. Heguri, N. H. Tu, K. K. Huynh, T. Fujita, T. Takahashi, K. Tanigaki, M. Chen, *Angew. Chem. Int. Ed.* 2014, 53, 4822.

[ii] Y. Tanabe, Y. Ito, K. Sugawara, D. Hojo, M. Koshino, T. Fujita, T. Aida, X. Xu, K. K. Huynh, H. Shimotani, T. Adschiri, T. Takahashi, K. Tanigaki, H. Aoki, M. Chen, *Adv. Mater.*, 2016, 28, 10304−10310.
